# Supplementary material for: Individual level analysis of digital proximity tracing for COVID-19 in Belgium highlights major bottlenecks
Source: Nat Commun. 2023 Oct 23;14:6717. doi: 10.1038/s41467-023-42518-6 (PMC10593825; doi:10.1038/s41467-023-42518-6)
Supplement: Supplementary file 1 — Supplementary Information [file 41467_2023_42518_MOESM1_ESM.pdf]

Individual level analysis of digital proximity tracing for COVID-19 in Belgium highlights major bottlenecks

Supplementary information

Authors: Caspar Geenen*, Joren Raymenants*, Sarah Gorissen, Jonathan Thibaut, Jodie McVernon, Natalie Lorent, Emmanuel André

* Contributed equally

Supplementary Tables

Supplementary table 1. Test indications and their description in the online test booking form. *Test indications evolved over the duration of the test and trace programme, in line with changing national guidelines. All test indications used throughout the extended study period are listed here with their identifier codes, implementation dates and exact wording in the English version of the online test booking form.*

**Identifier**	**Period implemented**	**Description in test booking form**
pos_self_test	from 27/04/2021	a positive self test
symptoms	before 25/08/2021	immediately after getting mild COVID-19 symptoms
	from 25/08/2021	mild COVID-19 symptoms (as soon as possible)
close_contact	before 25/08/2021	immediately (between 1 and 3 days) after a high-risk contact with a proven COVID-19 positive person
		7 days after a high-risk contact with a proven COVID-19 positive person
	25/08/2021 - 16/12/2021	a risk contact (preferably on days 1 to 3, with a second test on day 7)
	from 16/12/2021	a risk contact (see website for advised test dates)
return_abroad	before 27/04/2021	on day 1 after returning from a red zone abroad
		on day 7 after returning from a red zone abroad
		on day 14 after a return from or passing through India, Latin America or South Africa
	from 25/08/2021	arrival from a red zone (on days 1 and/or 7, depending on government's instructions after PLF (passenger locator form)
coronalert	before 25/08/2021	after getting a red screen on the CoronAlert app
	from 25/08/2021	a red screen on CoronAlert
extended_tracing	before 25/08/2021	by referral from the contact tracers of the KU Leuven who informed me about a confirmed infection in my student residence
	from 25/08/2021	referral from the contact tracers of the KU Leuven who informed me about a confirmed infection
start_internship	throughout study period	the start of my (para)medical internship
precaution	before 25/08/2021	no specific reason
	from 25/08/2021	a concern about a possible infection
medical_treatment	from 25/08/2021	a hospital admission or treatment, that requires preventive testing

Supplementary table 2. Model input parameters for comparing manual or digital contact tracing to case isolation only. *This table lists the parameters used to model the effect of different TTIQ strategies (test, trace, isolate, quarantine) on the effective reproduction number in our setting. The parameters were used as input for the tti package in R*[1,2]*. Baseline: no intervention. Isolation: case isolation only. DPT: case isolation with DPT. MCT: case isolation with MCT. DPT+MCT: case isolation with combined DPT and MCT*

Parameter	Explanation	Input value	Source
alpha	The probability of an asymptomatic infection	**0.2**	[3]
R	Effective reproduction number in fully vaccinated Delta/Omicron dominant population with moderate contact restrictions	**1.5**	The national R_{eff} (based on case numbers) started at 1.15 at the start of the main study period (18 October 2021), climbed to 1.28 at the start of November, decreased to a minimum of 0.69 at the start of December. With the arrival of omicron variant, it increased again to a new peak of 1.68 at the end of December, before declining again to 1.15 at the end of the study period[4]. The R_0 associated with the Delta and Omicron variants imply that a baseline R_{eff} of 1.5 or 2 can only be achieved with a combination of immunity, general contact restrictions, and barrier measures[5,6].
Kappa	Relative transmissibility of an asymptomatic individual compared to a symptomatic individual.	**0.35**	[3]
Eta	Probability that a contact is a household contact.	**0**	We do not differentiate between household and non-household contacts in this model.
nu	Relative risk of infection for a household contact compared to a community contact.	**1**	Not relevant, as we do not consider household contacts separately.
t_ds	Time delay from symptom onset to isolation in detected symptomatic person. [days]	Baseline: **100**<br>Other strategies: **2.34**	Sum of the mean delays between (1) symptom onset and sampling, and (2) sampling and PCR test result. (Supplementary Figure 4).
t_da	Time delay from symptom onset to isolation in detected asymptomatic person. [days]	**100**	Practically infinite delay, as we considered only symptomatic screening as index case detection strategy.
t_qcs / t_qhs	Time delay from symptomatic index cases's symptom onset to quarantine of	Baseline: **100**<br>Isolation: **100**<br>MCT: **4.60**<br>DPT: **3.52**	Sum of t_ds and estimate of the mean delay from case PCR test result to contact notification. This delay is

Parameter	Explanation	Input value	Source
	community/household contacts. [days]	MCT+DPT: **4.49**	determined for MCT by fitting a log-normal curve to the distribution of observed values. For DPT, it is determined with a log-normal curve as described in *Methods*. The delay for a combined DPT and MCT strategy is determined by calculating the probability that either a manual or digital notification is received, based on both lognormal curves.
t_qca / t_qha	Time delay from asymptomatic index cases's symptom onset to quarantine of community/household contacts. [days]	**100**	We considered only symptomatic screening as index case detection strategy.
t_q	Time delay from quarantined index cases's symptom onset to quarantine of contacts. [days]	Equal to t_qcs	Assumed equal to detection through symptomatic screening.
omega_c / omega_h / omega_q	The probability of being traced and quarantined given community / household / quarantine contact of a person.	Baseline: **0**<br>Isolation: **0**<br>MCT: **0.441**<br>DPT: **0.043**<br>MCT+DPT: **0.464**	As per the estimation in *Table 1*. No differentiation by contact type.
quarantine_ days	The number of days contacts are told to quarantine. [days]	**7**	Standard guidance in Belgium at the time.
rho_s	The probability of detection and isolation given symptomatic. Product of case acertainment and effectiveness of isolation to interrupt transmission (assumed to be 100).	Varied from **0.1 to 0.9** in 0.1 increments	Highly reliant on specific testing resources and willingness to test[7–9].
rho_a	Same as rho_s, but for asymptomatic individuals.	**0**	We considered only symptomatic screening and contact tracing as case detection strategies.
t_incubation	Mean incubation period. [days]	**4.2**	Average of incubation periods of the Delta and Omicron BA1 variants[10].
offset	Offset of infectiousness compared to symptoms onset. [days]	**-2.31**	Baseline model assumption[1], based on [11].
shape	Shape of the gamma distribution of infectious period.	**1.65**	Baseline model assumption[1], based on [11].
rate	Rate of the gamma distribution of infectious period.	**0.5**	Baseline model assumption[1], based on [11].

Parameter	Explanation	Input value	Source
stoch	Whether to run stochastic model with overdispersion.	**FALSE**	
Theta	Overdispersion parameter for negative binomial distribution.	**0.1** (only for sensitivity analysis with stochastic model)	Baseline model assumption[1], based on [11].
N_inf	Number of infections (or effective population size of infected individuals).	**20** (only for sensitivity analysis with stochastic model)	Common number of new daily infections in the university testing centre
N_iter	Number of iterations of stochastic model to run for each unique parameter value.	**100,000** (only for sensitivity analysis with stochastic model)	

Supplementary table 3. Results of sensitivity analyses when modelling the effective reproduction number. *Selected parameters were altered relative to the nominal model parameters in Supplementary table 2. This table lists which parameters were altered and the resulting modelled effect of each contact tracing strategy on the effective reproduction number, relative to case isolation only (columns 2 to 4). The last two columns show the effect of DPT and combined DPT/MCT, respectively, on the effective reproduction number, relative to MCT (with case isolation also implemented in each strategy).*

**Altered parameter(s)**	**Case isolation + DPT**	**Case isolation + MCT**	**Case isolation + DPT + MCT**	**Effect of DPT relative to MCT (given case isolation)**	**Effect of DPT+MCT relative to MCT (given case isolation)**
Model without alterations	1.06	1.58	1.63	0.11	1.08
Stochastic model	1.06	1.62	1.68	0.09	1.09
Stochastic model,<br>N_iter = 200,000	1.06	1.62	1.67	0.10	1.08
Lower limit (2.8%) of DPT success confidence interval, i.e.,<br>multiply all omega values with 0.65.	1.04	1.37	1.39	0.11	1.08
Upper limit (6.1%) of DPT success confidence interval, i.e.,<br>multiply all omega values with 1.42.	1.09	1.87	1.95	0.10	1.09
Lower limit (0.6) of DPT delay confidence interval, i.e., t_qcs, t_qhs, t_q =<br>2.97 (DPT)<br>4.45 (DPT+MCT)	1.07	1.58	1.64	0.11	1.09
Upper limit (2.2) of DPT delay confidence interval, i.e., t_qcs, t_qhs, t_q =<br>4.57 (DPT)<br>4.56 (DPT+MCT)	1.05	1.58	1.62	0.09	1.07
4-day delay from case PCR result to MCT notification, i.e.,<br>t_qcs = t_qhs = t_q =<br>6.34 (MCT)<br>6.07 (DPT/MCT)	1.06	1.35	1.41	0.17	1.18
alpha = 0.5	1.06	1.61	1.66	0.10	1.08
alpha = 0.1	1.06	1.58	1.63	0.11	1.08
R = 1.2	1.06	1.58	1.63	0.11	1.08
R = 5	1.06	1.58	1.63	0.11	1.08
Kappa = 2	1.06	1.62	1.67	0.10	1.08
Kappa = 0.2	1.06	1.58	1.63	0.11	1.08
t_ds = 4 (all except baseline strategy)	1.15	2.46	2.57	0.10	1.08

t_ds = 1 (all except baseline strategy)	1.03	1.25	1.27	0.11	1.09
Add 1 day to values of t_qcs, t_qhs and t_q	1.05	1.46	1.50	0.11	1.10
Subtract 1 day from values of t_qcs, t_qhs and t_q	1.07	1.68	1.73	0.10	1.07
Multiply all omega values with 0.5	1.03	1.28	1.30	0.11	1.08
Multiply all omega values with 1.5	1.09	1.93	2.01	0.10	1.09
t_incubation = 2	1.06	1.53	1.58	0.12	1.10
t_incubation = 6	1.05	1.50	1.54	0.10	1.08
offset = 0	1.01	1.08	1.08	0.10	1.09
offset = -4	1.15	2.36	2.48	0.11	1.09

Supplementary Figures

Supplementary Figure 1. Exclusion chart for the infection risk analysis by test indication for the main study period (18 October 2021 - 9 January 2022). *See Methods for the exact questions asked, and Supplementary table 1 for a full list of test indications implemented throughout the study period and their grouping for this analysis.*

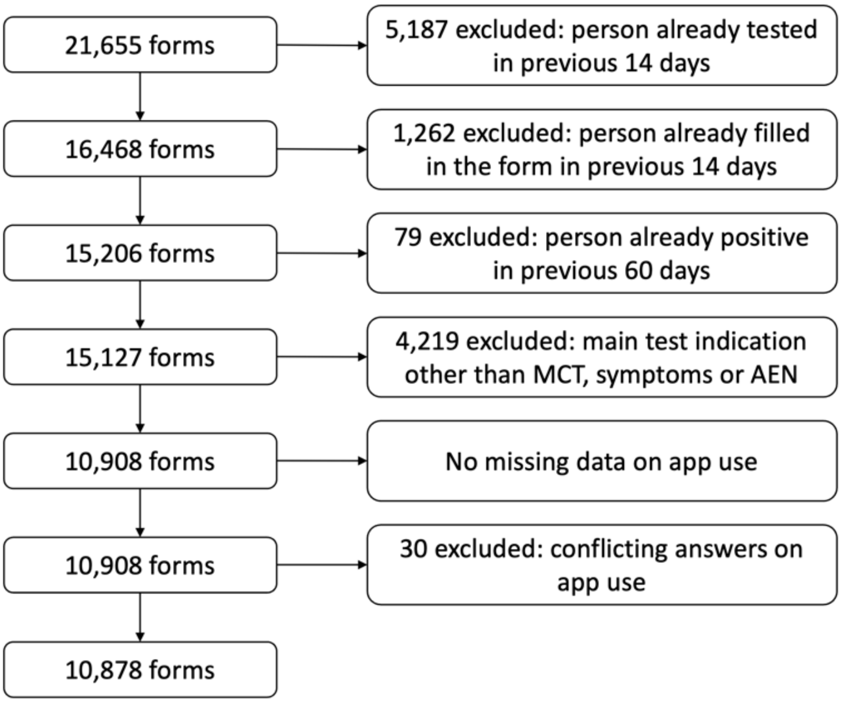

Supplementary Figure 2. Exclusion chart for the infection risk analysis by test indication for the extended study period (1 February 2021 until 21 March 2022). *See Methods for the exact questions asked, and Supplementary table 1 for a full list of test indications implemented throughout the study period and their grouping for this analysis.*

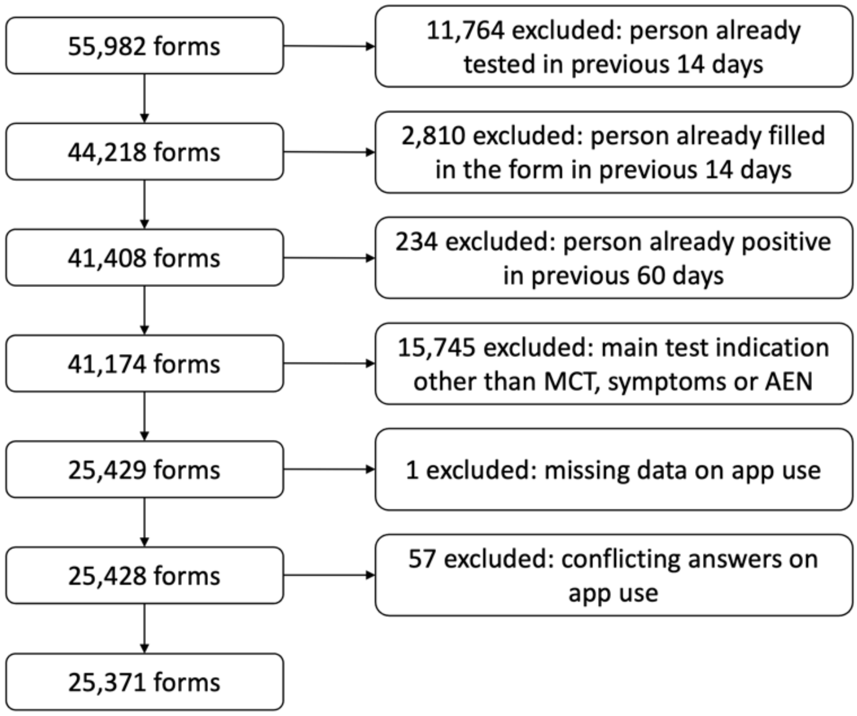

Supplementary figure 3. Number of tests, infection risk and risk ratio by self-reported test indication for persons undergoing a test at the university test centre in the extended study period (1 February 2021 - 21 March 2022). *Persons (n=25,371 individual test bookings) are grouped according to self-reported main test indication: manually traced close contact, receipt of an automated exposure notification (AEN), or symptoms suggestive for COVID-19. The exclusion chart is shown in Supplementary Figure 2. In panel a, each group is subdivided according to app use and AEN receipt. For each subgroup, the number of infected and uninfected persons, the number of persons lost to follow-up, and the infection risk (the proportion subsequently diagnosed with COVID-19) are listed. Error bars indicate the observed infections risks with 95% confidence intervals. In addition, the risk ratio is shown relative to non-app users who were manually traced or had suggestive symptoms. In panel b, each group is subdivided according to outcome: infected, not infected or lost to follow-up. For each subgroup, the number of app users with and without an AEN is indicated, as well as the numbers of people not using the app. The observed rate of AEN receipt amongst app users is shown for each subgroup, with error bars indicating 95% confidence intervals. Relative risks are calculated compared to a control group of persons attending for suggestive symptoms, but not infected with COVID-19. We consider this control group a surrogate for the average rate of AEN receipt (justified or not) in the population. Reassuringly, both infected symptomatic persons and those with a manually traced close contact had a similarly increased rate of AEN receipt. Asterisks indicate significant differences using a two-sided Pearson's chi-squared test, not adjusted for multiple comparisons.*

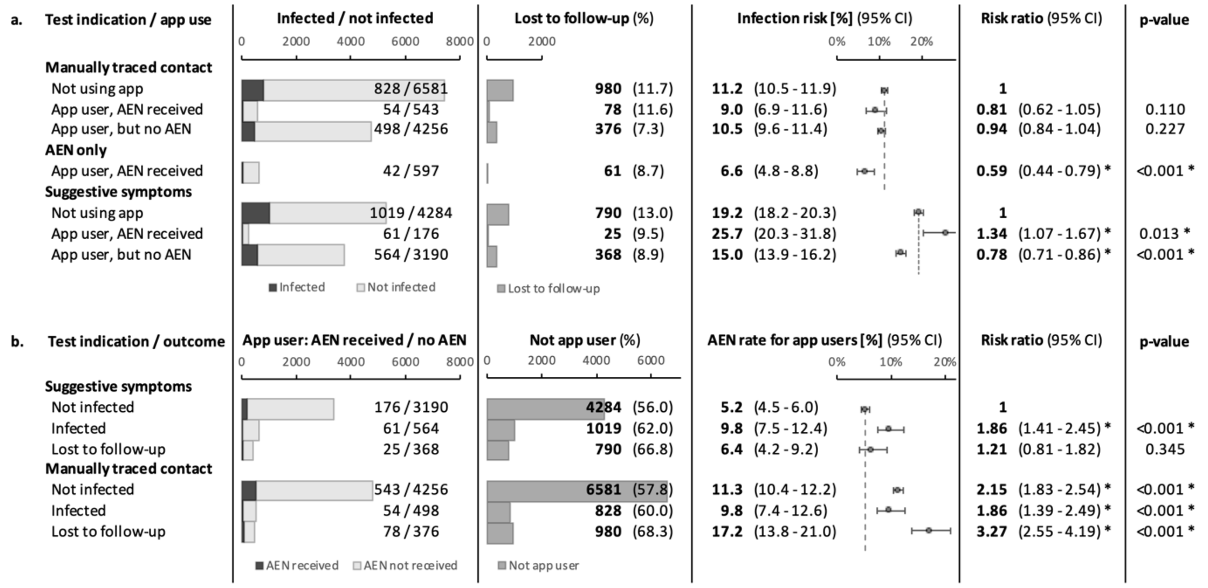

Supplementary Figure 4. Schematic representation of delays in the digital notification cascade during the main study period. *The mean delay is shown for each step, with 95% confidence intervals between brackets. Symptom onset was assessed during contact tracing for persons who were diagnosed through symptomatic screening (n=341 cases identified through symptomatic screening; no symptom onset date: 104 cases). The delay between sampling and test result was assessed for persons testing positive at our test centre (n=1,715 cases). Estimated delays from positive result to key upload (n=560 interviewed active users with known exact interview time) and contact notification (n=162 tests booked by a contact when both the contact and the case are active users) are shown in italic. The estimated values were modelled by fitting a cumulative log-normal function to the data, as described in Methods and Results.*

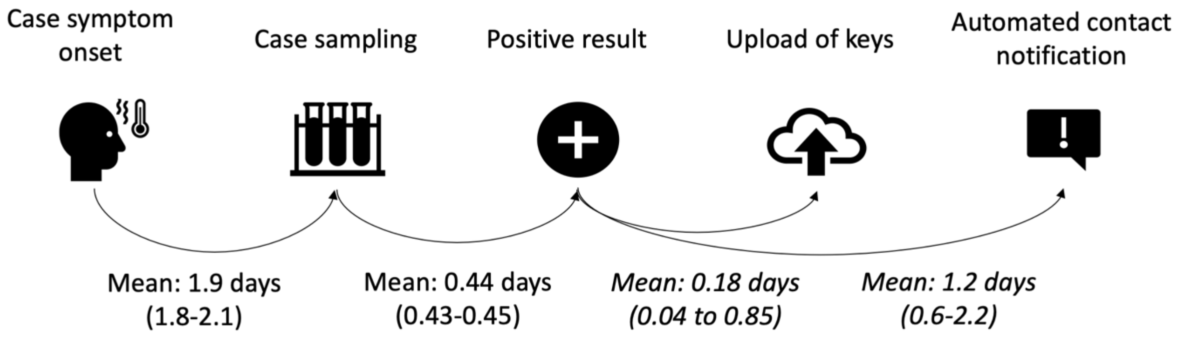

Supplementary Figure 5. Modelled effect on the effective reproduction number (R_{eff}) of different case isolation and contact quarantine strategies in our setting. *Panel a shows how the modelled effective reproduction number decreases almost linearly with the proportion of cases identified through symptomatic screening. Panel b shows the effect of each strategy on R_{eff} relative to case isolation only.*

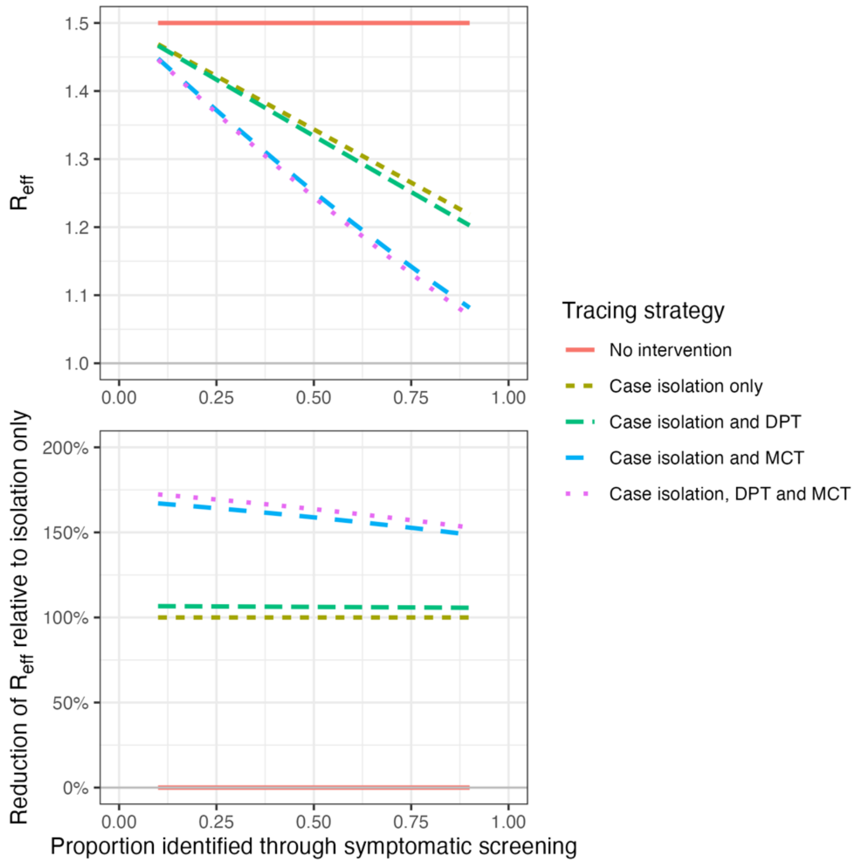

Supplementary Figure 6. Incidence and additional measures of MCT and DPT performance in the extended study period. *Panel a shows the weekly number of confirmed cases amongst Leuven students and the Belgian population. Panels b and d indicate the total number of manually traced contacts and the number of contacts per case (n=6,856 confirmed cases), respectively. Panel c shows the proportion of persons who received an automated digital notification (AEN), amongst those attending the test centre for a manually traced exposure (n=14,194 MCT test bookings). Dots and error bars in panel c show weekly observed proportions with 95% confidence intervals. Grey dots in panel d show individual data points. Vertical black lines show the beginning and end of the main study period. Coloured lines with bands show estimates obtained using local polynomial regression curves with 95% confidence intervals. A change in app configuration, intended to reduce the number of notified contacts, is indicated with a blue vertical line.*

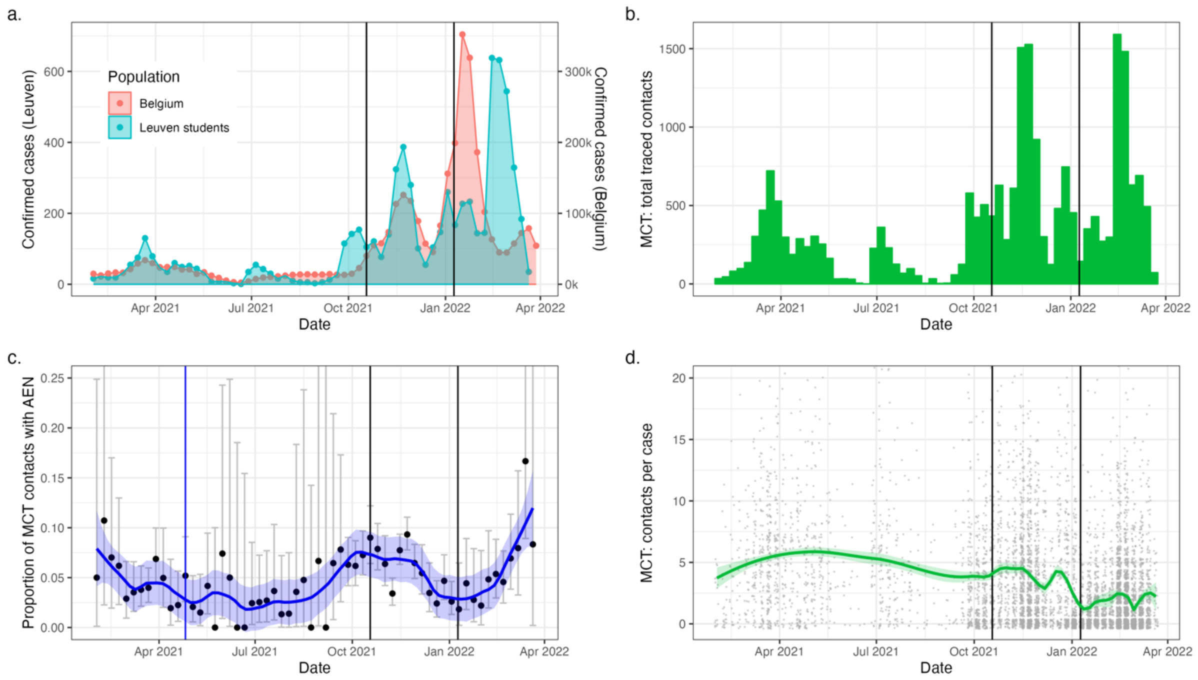

References

1. Grantz, K. H. *et al.* Maximizing and evaluating the impact of test-trace-isolate programs: A modeling study. *PLoS Med* **18**, e1003585 (2021).
2. McGowan, L. D., Grantz, K., Lee, E. & Lessler, J. HopkinsIDD/tti: First release of TTI. (2020) doi:10.5281/ZENODO.4012424.
3. Buitrago-Garcia, D. *et al.* Occurrence and transmission potential of asymptomatic and presymptomatic SARS-CoV-2 infections: A living systematic review and meta-analysis. *PLoS Med* **17**, e1003346 (2020).
4. Daily SARS-CoV2 Re Values for select countries. https://github.com/covid-19-Re/dailyRe-Data (2023).
5. Liu, Y. & Rocklöv, J. The reproductive number of the Delta variant of SARS-CoV-2 is far higher compared to the ancestral SARS-CoV-2 virus. *J Travel Med* **28**, (2021).
6. Liu, Y. & Rocklöv, J. The effective reproductive number of the Omicron variant of SARS-CoV-2 is several times relative to Delta. *J Travel Med* **29**, 1–4 (2022).
7. Irons, N. J. & Raftery, A. E. Estimating SARS-CoV-2 infections from deaths, confirmed cases, tests, and random surveys. *Proc Natl Acad Sci U S A* **118**, e2103272118 (2021).
8. Colman, E., Puspitarani, G. A., Enright, J. & Kao, R. R. Ascertainment rate of SARS-CoV-2 infections from healthcare and community testing in the UK. *J Theor Biol* **558**, 111333 (2023).
9. Mathieu, E. *et al.* Coronavirus Pandemic (COVID-19). https://ourworldindata.org/coronavirus (2020).
10. Du, Z. *et al.* Shorter serial intervals and incubation periods in SARS-CoV-2 variants than the SARS-CoV-2 ancestral strain. *J Travel Med* **29**, (2022).
11. He, X. *et al.* Author Correction: Temporal dynamics in viral shedding and transmissibility of COVID-19. *Nat Med* **26**, 1491–1493 (2020).
