# Supplementary material for: Individual level analysis of digital proximity tracing for COVID-19 in Belgium highlights major bottlenecks
Source: Nat Commun. 2023 Oct 23;14:6717. doi: 10.1038/s41467-023-42518-6 (PMC10593825; doi:10.1038/s41467-023-42518-6)
Supplement: Supplementary file 3 — Description of Additional Supplementary Files [file 41467_2023_42518_MOESM3_ESM.pdf]

Description of Additional Supplementary Files

File Name: Supplementary Data
Description: Individual level data required to reproduce the analyses in this article. A data dictionary is provided in the first tab. For privacy reasons, only summary statistics are provided for age data.
