# Supplementary material for: Individual level analysis of digital proximity tracing for COVID-19 in Belgium highlights major bottlenecks
Source: Nat Commun. 2023 Oct 23;14:6717. doi: 10.1038/s41467-023-42518-6 (PMC10593825; doi:10.1038/s41467-023-42518-6)
Supplement: Supplementary file 5 — Reporting Summary [file 41467_2023_42518_MOESM5_ESM.pdf]

nature portfolio

Corresponding author(s): Caspar Geenen
Last updated by author(s): Oct 6, 2023

Reporting Summary

Nature Portfolio wishes to improve the reproducibility of the work that we publish. This form provides structure for consistency and transparency in reporting. For further information on Nature Portfolio policies, see our Editorial Policies and the Editorial Policy Checklist.

Statistics

For all statistical analyses, confirm that the following items are present in the figure legend, table legend, main text, or Methods section.

n/a	Confirmed	
☐	☒	The exact sample size (*n*) for each experimental group/condition, given as a discrete number and unit of measurement
☐	☒	A statement on whether measurements were taken from distinct samples or whether the same sample was measured repeatedly
☐	☒	The statistical test(s) used AND whether they are one- or two-sided<br>*Only common tests should be described solely by name; describe more complex techniques in the Methods section.*
☐	☒	A description of all covariates tested
☐	☒	A description of any assumptions or corrections, such as tests of normality and adjustment for multiple comparisons
☐	☒	A full description of the statistical parameters including central tendency (e.g. means) or other basic estimates (e.g. regression coefficient) AND variation (e.g. standard deviation) or associated estimates of uncertainty (e.g. confidence intervals)
☐	☒	For null hypothesis testing, the test statistic (e.g. *F*, *t*, *r*) with confidence intervals, effect sizes, degrees of freedom and *P* value noted<br>*Give P values as exact values whenever suitable.*
☒	☐	For Bayesian analysis, information on the choice of priors and Markov chain Monte Carlo settings
☒	☐	For hierarchical and complex designs, identification of the appropriate level for tests and full reporting of outcomes
☒	☐	Estimates of effect sizes (e.g. Cohen's *d*, Pearson's *r*), indicating how they were calculated

Our web collection on statistics for biologists contains articles on many of the points above.

Software and code

Policy information about availability of computer code

Data collection	For data collection we used Go.Data, a contact tracing tool developed by the World Health Organisation. Go.Data version: 2.37.0 - build 2105111528. Go.Data was integrated with custom-built appointment management, contact listing, and laboratory result modules.
Data analysis	Data analysis was performed using R scripts specifically written for this study in R version 4.2.2, using the following packages: tidyverse (2.0.0), epitools (0.5-10.1), lubridate (1.9.2), patchwork (1.1.2), DescTools (0.99.48), tti (0.1.0), ggtext (0;1.2), gridExtra (2.3), readxl (1.4.2), ggrepel (0.9.3), fitdistrplus (1.1-8), investr (1.4.2), and MASS (7.3-58.3). The scripts are available online: https://github.com/c-geenen/DPT-leuven

For manuscripts utilizing custom algorithms or software that are central to the research but not yet described in published literature, software must be made available to editors and reviewers. We strongly encourage code deposition in a community repository (e.g. GitHub). See the Nature Portfolio guidelines for submitting code & software for further information.

Data

Policy information about availability of data

All manuscripts must include a data availability statement. This statement should provide the following information, where applicable:

- Accession codes, unique identifiers, or web links for publicly available datasets
- A description of any restrictions on data availability
- For clinical datasets or third party data, please ensure that the statement adheres to our policy

The individual-level data underlying the main analyses in this study are provided in the Supplementary Data file. The raw age data are available under restricted

access for privacy reasons. Access to age data can be obtained by request to the corresponding author (C.G.), who will respond within 4 weeks. There must be a demonstrable affiliation with an academic or health institution, a legitimate epidemiological question, and a commitment not to attempt to de-anonymise.

Research involving human participants, their data, or biological material

Policy information about studies with human participants or human data. See also policy information about sex, gender (identity/presentation), and sexual orientation and race, ethnicity and racism.

Reporting on sex and gender	For participants undergoing testing at our test centre, sex was determined based on the Belgian national register, which allows only binary options. The proportion of female cases was 54.1% (missing data: 6.4%).
Reporting on race, ethnicity, or other socially relevant groupings	We did not collect race, ethnicity or other social variables.
Population characteristics	The mean age of cases was 21.6 years (standard deviation: 3.2 years; missing data: 8.1%).
Recruitment	The study specifically targeted young adults in higher education, possibly resulting in a bias towards persons with higher-than-average willingness and ability to engage in contact tracing. We do not expect this to influence our estimates of technical sensitivity or infection risk, which should be independent of app uptake and the proportion consenting to contact tracing.
Ethics oversight	The study protocol was approved by the Ethics Committee Research UZ/KU Leuven (reference number: S64919). Informed consent was waived as the data gathered did not exceed what was required for the purpose of safeguarding public health.

Note that full information on the approval of the study protocol must also be provided in the manuscript.

Field-specific reporting

Please select the one below that is the best fit for your research. If you are not sure, read the appropriate sections before making your selection.

☒ Life sciences ☐ Behavioural & social sciences ☐ Ecological, evolutionary & environmental sciences

For a reference copy of the document with all sections, see nature.com/documents/nr-reporting-summary-flat.pdf

Life sciences study design

All studies must disclose on these points even when the disclosure is negative.

Sample size	No power analysis was performed, because the study size was a direct result of the number of cases and contacts during the study period, which was chosen as broadly as possible. The study period ran from 18 October 2021 – when we started systematically asking cases whether they triggered digital notifications – to 9 January 2022, when government-mandated testing for all close contacts was abandoned. The obtained study size is reflected in the confidence intervals of the results.
Data exclusions	For the infection risk analysis, we included all students who filled out an online test booking form and indicated a manually traced contact, suggestive symptoms, or an AEN as the main test indication. Students were excluded if they had already been identified as infected in the previous 60 days. To avoid selection bias and ambiguous test indications, we also excluded students who had already booked or undergone a test in the preceding 14 days. Finally, we excluded persons who indicated an AEN as the test indication but answered “no” in the same questionnaire, when asked whether an AEN was received.<br><br>For the analysis of the notification cascade, we started from all infected students referred to our contact tracing team for a positive test in the study period. If the contact tracer considered the positive test as likely due to a previous infection, or if data on app use was missing, the case was excluded. When determining success rates and delays in the AEN triggering step, we also excluded cases who could not be reached for an interview, cases interviewed only before their PCR test result, and cases with missing information on whether they triggered notifications. Contacts of cases who triggered AENs were included in the analysis if the contact filled out an online PCR test booking form for our test centre, 0 to 14 days after the case PCR result was reported. Contacts were excluded if they already had a positive test up to 60 days before the case. If a contact was exposed to several cases, only the first reported case-contact pair was retained.
Replication	The findings of this observational study were not checked for reproducibility.
Randomization	Randomization was not applicable in this observational study. Participants were allocated to groups based on the described criteria.
Blinding	Blinding was not relevant to this observational study.

Reporting for specific materials, systems and methods

We require information from authors about some types of materials, experimental systems and methods used in many studies. Here, indicate whether each material, system or method listed is relevant to your study. If you are not sure if a list item applies to your research, read the appropriate section before selecting a response.

Materials & experimental systems

n/a	Involved in the study
☒	☐ Antibodies
☒	☐ Eukaryotic cell lines
☒	☐ Palaeontology and archaeology
☒	☐ Animals and other organisms
☒	☐ Clinical data
☒	☐ Dual use research of concern
☒	☐ Plants

Methods

n/a	Involved in the study
☒	☐ ChIP-seq
☒	☐ Flow cytometry
☒	☐ MRI-based neuroimaging
